# Feeding patterns reprogram a gut microbial virulence–iron–quorum sensing functional axis linked to atherosclerotic risk

**DOI:** 10.3389/fmicb.2025.1751844

**Published:** 2026-01-21

**Authors:** He Zhang, Keyu Chen, Renjin Chen, Erteng Jia

**Affiliations:** 1 School of Life Sciences, Xuzhou Medical University, Xuzhou, China; 2 Thoracic Surgery Laboratory, The First College of Clinical Medicine, Xuzhou Medical University, Xuzhou, China

**Keywords:** circadian misalignment, feeding rhythm, gut microbial virulence, iron acquisition, Type III/VI secretion systems

## Abstract

The feeding rhythm is a major temporal regulator of metabolic physiology, yet its impact on microbiome-derived functional traits relevant to cardiometabolic disease remains insufficiently understood. Our previous work demonstrated that ad libitum, daytime-restricted, and nighttime-restricted feeding produce markedly different atherosclerotic outcomes in Apoe^−^/^−^ mice, indicating that the feeding rhythm acts as a modifiable determinant of atherogenic susceptibility. Here, we used shotgun metagenomics to profile risk-associated microbial functional modules—including Type III and Type VI secretion systems (T3SS/T6SS), siderophore-based iron acquisition pathways, quorum-sensing (QS) regulators, and antimicrobial resistance determinants—across feeding regimens. The feeding rhythm induced pronounced functional segregation independent of *α*-diversity, which was consistent with selective functional reprogramming rather than taxonomic restructuring. Daytime feeding, which is misaligned with the murine active phase, is associated with coordinated enrichment of the T3SS/T6SS, iron uptake, and QS pathways, forming a tightly interconnected “virulence–iron–QS–ARG” functional consortium. In contrast, circadian-aligned nighttime feeding resulted in attenuated virulence orientation and enhanced metabolic-cooperative signatures. Network inference further revealed strong coactivation of virulence secretion, iron mobilization, and QS modules under circadian misalignment. These findings show that the feeding rhythm modulates atherogenic susceptibility not only through host metabolism but also by remodeling gut microbial functional capacities, highlighting microbial functional ecology as an integral component of diet–host interactions.

## 1 Introduction

Atherosclerotic cardiovascular disease (ASCVD) is the most common cause of death worldwide and accounts for about one-third of all deaths each year, making it a major public health problem (Virani et al., 2020). The development of ASCVD involves both non-modifiable factors, such as age and genetics, and modifiable factors, including diet, high blood pressure, and obesity (Virani et al., 2021; Ibanez et al., 2021; Libby et al., 2019). These factors lead to lipid buildup in blood vessels, long-term inflammation, and plaque formation, which can cause serious cardiovascular events (Ruscica et al., 2018; Espirito-Santo et al., 2023). Statins and blood-pressure-lowering drugs can slow disease progression, but ASCVD remains the leading cause of death worldwide (Ference et al., 2019; Mach, 2020).

Dietary patterns are an important modifiable risk factor. In China, unhealthy diets are the main contributor to cardiovascular disease incidence and mortality (Yusuf et al., 2004; Roth et al., 2020; Vaduganathan et al., 2022). Healthy dietary patterns, such as the Mediterranean diet and the DASH diet, can reduce ASCVD risk by improving metabolic measures (Esposito et al., 2015). The effects of diet extend beyond nutrient composition. Dietary patterns dynamically reshape gut microecology to modulate metabolic homeostasis (Luo et al., 2025). The molecular mechanisms linking specific dietary patterns to ASCVD are still not fully understood, especially the role of gut microbial functional traits rather than microbial composition alone.

In recent years, research on the gut-cardiovascular axis has advanced, and the gut microbiota has been recognized as a key link between diet and ASCVD development. Previous studies show that gut microbiota imbalance is not only a local ecological change. It also promotes atherosclerosis through low-grade inflammation, endothelial damage, metabolic disturbance, and microbe-derived functional factors, such as antibiotic resistance genes, quorum-sensing systems, and virulence factors (Beutler and Rietschel, 2003; Zhu et al., 2022; Bartolomaeus et al., 2019). As an environmental factor that closely interacts with diet, the composition, metabolites, and functional genes of the gut microbiota are strongly influenced by dietary patterns (Chen et al., 2024; Bolte et al., 2021; Ma et al., 2025). Most current studies focus on changes in microbial composition or metabolites, such as trimethylamine N-oxide and short-chain fatty acids. However, how dietary patterns affect ASCVD by regulating functional components of the gut microbiota, including quorum-sensing genes and virulence factors, has not been studied in a systematic way.

As previously reported in our earlier study using the same feeding paradigm (Zhang et al., 2025a), daytime feeding was associated with reduced fluorescence intensities of TGR5 and ABCA1, together with increased signals of NF-κB, IL-6, IL-1β, and TNF-*α*, indicating impaired cholesterol efflux and enhanced vascular inflammation. Differentially abundant metabolites (DAMs) were identified by pairwise comparisons using OPLS-DA (VIP > 1) together with univariate tests (|log₂ fold change| > 1, *p* < 0.05). A total of 31 DAMs were detected between the ad libitum and daytime feeding groups, 111 between the ad libitum and nighttime feeding groups, and 79 between the daytime and nighttime feeding groups. Notably, taurolithocholic acid (TLCA) showed a robust inverse correlation with aortic lesion burden and was markedly reduced in the daytime feeding group (Zhang et al., 2025a).

Metagenomic sequencing combined with bioinformatics analysis allows not only the study of microbial community structure and metabolic potential, but also accurate analysis of functional genes, such as resistance genes, quorum-sensing genes, and virulence factors. This approach overcomes the limits of traditional microbial studies that focus mainly on community composition. It also provides technical support for detailed analysis of the relationship between diet and ASCVD through microbial functional features. Therefore, this study uses metagenomic and bioinformatics technologies to systematically explore the associations between dietary patterns and antibiotic resistance genes (ARGs)/quorum-sensing (QS)/virulence factors (VF) as well as their regulatory mechanisms in ASCVD. This study aims to fill the current research gap, improve the theoretical system of the “gut–cardiovascular axis,” and provide a scientific basis for the development of new ASCVD prevention and treatment strategies.

## 2 Materials and methods

### 2.1 Experimental animals, design, and diet

Female Apoe^−^/^−^ mice (6 weeks old) were obtained from Jiangsu GemPharmatech Co., Ltd., and acclimated for 1 week before experimentation. All procedures were approved by the Institutional Animal Care and Use Committee (IACUC) of Xuzhou Medical University and conformed to national laboratory animal welfare guidelines. A total of 54 age-matched mice were randomly assigned to three feeding regimens (n = 18/group): ad libitum feeding, daytime feeding, and nighttime feeding.

The mice were housed at a controlled temperature (22 ± 2 °C) and humidity (50% ± 10%) with a 12-h light/12-h dark cycle. To preserve internal circadian phase integrity, lights were scheduled from Zeitgeber time (ZT) 8 to ZT20. All the groups were fed a high-fat, high-cholesterol diet (HFHCD) for 14 consecutive weeks. The AL group received unrestricted food access across the 24-h cycle, whereas the NTF and DTF groups were restricted to food access exclusively during the dark phase (ZT20–ZT24, ZT0–ZT8) or light phase (ZT8–ZT20), respectively. Fresh colonic content samples were collected during the final week at matched circadian time points to avoid confounding temporal bias.

### 2.2 Sample collection, DNA extraction, and metagenomic library preparation

Fresh colonic content samples were rapidly collected under aseptic conditions, snap-frozen in liquid nitrogen, and stored at −80 °C until processing. Total microbial DNA was extracted using the QIAamp Fast DNA Stool Mini Kit (Qiagen, Germany) according to the manufacturer’s protocol, which included bead-beating–based mechanical lysis to ensure recovery of gram-positive organisms. The DNA concentration and purity were quantified with a Qubit dsDNA HS Assay Kit, and samples with OD₂₆₀_/_₂₈₀ ratios ranging from 1.8 to 2.2 were used for library construction.

The sequencing libraries were prepared via the Illumina TruSeq DNA PCR-free Library Preparation Kit. Indexed paired-end 150 bp libraries were sequenced on the Illumina NovaSeq 6,000 platform to obtain ≥10 Gb of clean data per sample, ensuring high-depth functional profiling.

### 2.3 Quality control, assembly, and gene catalog construction

Adapter trimming and removal of low-quality reads (Q < 20; minimum length 50 bp) were performed using fastp v0.23.2. Across all samples, raw sequencing reads showed high base-call accuracy. Q30 values ranged from 95.7 to 97.7% (mean ≈ 96.8%, Supplementary Table S1). Host-derived reads were removed by alignment to the mouse reference genome (GRCm39) using Bowtie2 v2.5.1 in very sensitive mode (Bolger et al., 2014). High-quality reads were assembled *de novo* using MEGAHIT v1.2.9 with a minimum contig length of 500 bp (Hyatt et al., 2010). Assembly quality was high. Contig N50 values ranged from 1,612 to 15,563 bp and were most often between 3,000 and 6,000 bp (Supplementary Table S1). Open reading frames were predicted using Prodigal v2.6.3 in metagenomic mode. A nonredundant gene catalog was then built using CD-HIT v4.8.1 with 95% sequence identity and 90% coverage. Gene abundance tables were generated by mapping reads back to the gene catalog using Salmon in quasi-mapping mode.

### 2.4 Taxonomic profiling

Taxonomic composition was inferred via MetaPhlAn4 with the CHOCOPhlAn 2023 marker gene database. Relative abundances were calculated at the phylum-to-species level. For microbial contributions to decomposition functional traits, StrainPhlAn and HUMAnN3 were used to resolve strain-level associations.

### 2.5 Functional annotation of virulence-associated modules

To profile virulence-associated functional traits specifically, annotated protein sequences within the nonredundant gene catalog were queried against the following (Table 1).

**Table 1:** Summary of functional annotation workflows used in this study.

Functional category	Database(s)	Tool/Software
Type III & VI secretion systems	VFDB (core), SecReT6	DIAMOND
Siderophore-mediated iron uptake & heme acquisition	IronBase, BacMet v2, KEGG BRITE (iron modules)	DIAMOND
Quorum-sensing regulators (LuxS, Lsr, Agr, Com systems)	QS-DB, MetaCyc signaling pathways	DIAMOND, HMMER
Antimicrobial resistance determinants (ARGs)	CARD, Resfams HMMs	CARD, Resfams HMMs

### 2.6 Quantification of secretion, iron, QS, and ARG indices

For comparative analysis, modules were aggregated into composite indices: Virulence Secretion Index (VSI) = normalized abundance of Type III secretion system (T3SS) + Type VI secretion system (T6SS) components; Iron Uptake Index (IUI) = siderophore biosynthesis + transport + heme utilization genes; QS Activation Score = lux-R/S, LsrK, Agr, Com, Rpf, and PDE-linked signaling genes; and ARG Burden Score = efflux pump + target protection + enzymatic inactivation pathways. Abundances were normalized via centered-log ratio (CLR) transformation before multivariate modeling.

### 2.7 Diversity and differential abundance analyses

*α*-Diversity: Shannon (richness + evenness) and Simpson (evenness-focused) indices were calculated via QIIME2 (v2022.11) on the basis of functional gene relative abundances. Statistical differences between feeding groups (ad libitum, daytime, and nighttime feeding) were tested via the Kruskal–Wallis H test, followed by Dunn’s *post hoc* correction to account for multiple comparisons.

*β*-Diversity: Principal coordinate analysis (PCoA) was performed via the Bray–Curtis dissimilarity matrix (derived from functional gene profiles) to visualize intergroup functional clustering. Permutational multivariate analysis of variance (PERMANOVA) with 999 permutations (via the vegan package in R v4.2.2) was used to assess significant differences in *β*-diversity among groups.

### 2.8 Correlation and network analyses

#### 2.8.1 Bivariate correlations

Spearman’s rank correlation coefficient was used to evaluate associations between microbial virulence secretion systems (T3SS, T6SS, Type VII secretion system (T7SS)) and the aortic atherosclerotic lesion area; secretion systems/iron metabolism pathways and plasma inflammatory cytokines (NF-κB, IL-1β, IL-6, TNF-*α*) or bile acid (BA) species; pathogen–host interaction (PHI) phenotypes; and host inflammatory/BA profiles. Correlation heatmaps were generated via the corrplot package in R, with significance annotated as *p* < 0.05 (*), *p* < 0.01 (**), and *p* < 0.001 (***).

#### 2.8.2 Functional co-occurrence network

SparCC correlation (implemented in the SparCC R package) was used to construct a functional co-occurrence network, minimizing compositional bias in the metagenomic data. Nodes represent functional modules (secretion systems, VF categories, ARG mechanisms, QS regulators, PHI phenotypes, iron metabolism indices, and the integrated virulence secretion index). Node size was proportional to the mean relative abundance of each module; edge width reflected the absolute correlation strength; edge color indicated positive (red) or negative (blue) correlations. Only significant correlations (FDR-adjusted p < 0.05) were retained for network visualization.

### 2.9 Redundancy analysis (RDA) and multivariate modeling

RDA was performed via the vegan package in R to quantify the relationships between microbial functional risk profiles (explanatory variables: T3SS, T6SS, iron acquisition, and ARGs) and feeding regimens (response variable: group identity). The functional traits are represented as vectors, and 95% confidence intervals (CIs) are plotted as ellipses to visualize group-specific clustering.

MaAsLin2 Modeling: Multivariate Association with Linear Models 2 (MaAsLin2) was used to estimate the effect of feeding rhythm on virulence-associated functional terms, adjusting for potential confounders (e.g., microbial taxonomic richness). Effect sizes (coefficient direction and magnitude) were reported to quantify group-level differences in functional enrichment.

### 2.10 Statistical analysis

All analyses were conducted in R (v4.2.2) via the following packages: vegan (diversity/ordination), ggplot2 (visualization), corrplot (correlations), and MaAsLin2 (multivariate modeling). A nominal *p* < 0.05 was considered significant for differential abundance and correlation analyses to reduce false-positive results. For the three-group (ad libitum, daytime, and nighttime) differential analyses of the relative abundances of the T3SS, T6SS, T7SS, and VSI, as well as iron metabolism-related functions (iron uptake, iron storage, iron oxidation, iron reduction, and iron gene regulation), nonparametric statistical methods were employed, specifically the Kruskal–Wallis H test for overall intergroup difference assessment followed by Dunn’s *post hoc* test with Benjamini–Hochberg false discovery rate (FDR) correction for pairwise difference identification.

## 3 Results

### 3.1 Feeding regimens reshaped the global microbial functional ecosystem

**Figure 1:**
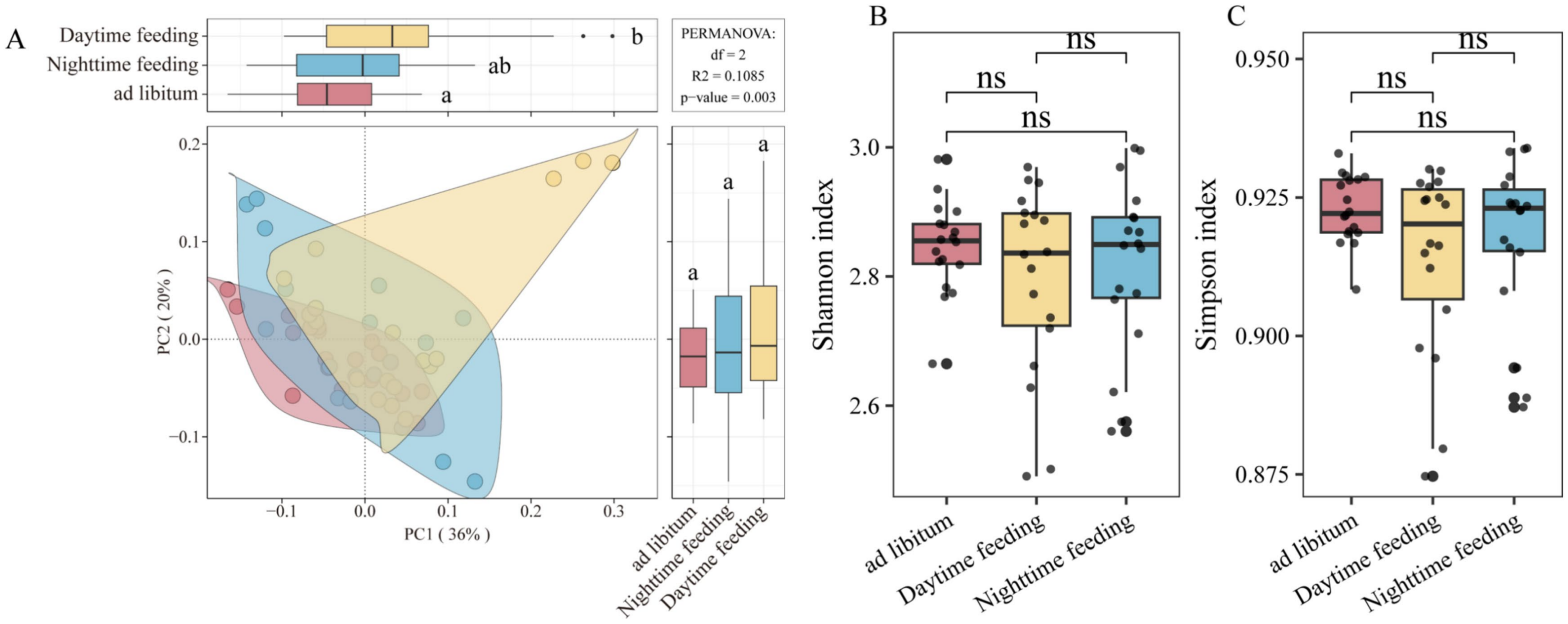
Feeding rhythm changes the global microbial functional structure. **(A)** Principal coordinate analysis of microbial functional profiles from colonic content metagenomic data among the ad libitum, nighttime feeding, and daytime feeding groups. **(B,C)** Boxplots of *α*-diversity indices (Shannon and Simpson) calculated from functional gene relative abundances in colonic content samples. ns, not significant.

To test whether time-restricted feeding changes overall microbial function, we performed PCoA based on Bray–Curtis dissimilarity. Samples from the three feeding regimens (ad libitum, daytime-restricted, and nighttime-restricted) separated clearly, showing that functional profiles differed among groups (PERMANOVA R^2^ = 0.082, *p* = 0.005; Figure 1A). At the same time, Shannon and Simpson diversity indices did not differ among groups (*p* > 0.05; Figures 1B,C). This result shows that functional differences were due to changes in composition and not changes in overall functional diversity.

### 3.2 Daytime feeding selectively enriches risk-oriented microbial functional signatures

**Figure 2:**
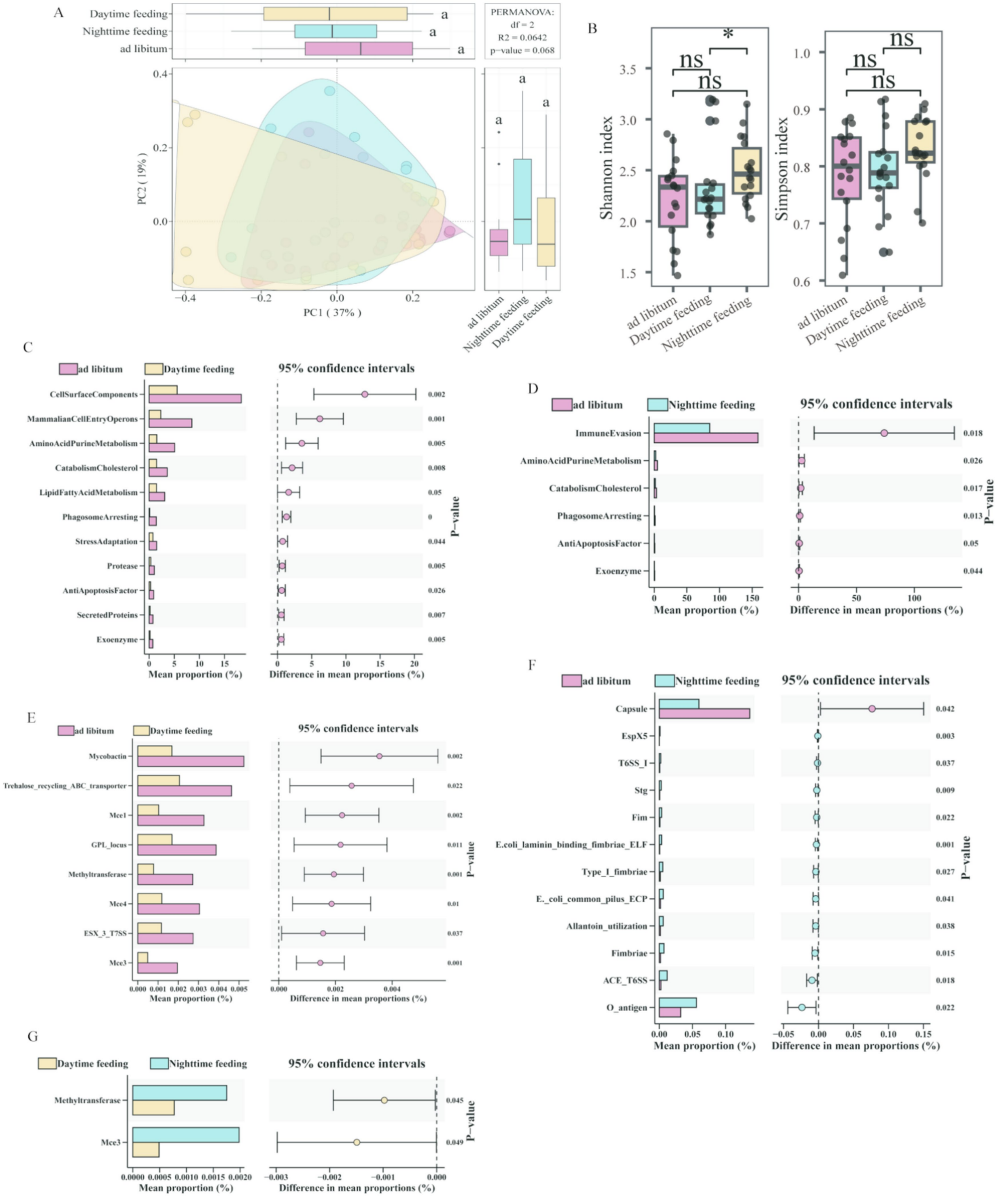
Diversity and differential abundance of virulence factor profiles among feeding patterns. **(A)** Principal coordinate analysis of virulence factor functional profiles from colonic content metagenomic data across the *ad libitum*, daytime feeding, and nighttime feeding groups. **(B)** Boxplots of α-diversity indices (Shannon and Simpson) based on virulence factor annotations from colonic content samples. **(C–G)** Differential enrichment of VFDB functional categories between feeding patterns. *p < 0.05, **p < 0.01, ***p < 0.001; ns, not significant.

To characterize functional changes associated with this reorganization, we quantified virulence-related PCoA revealed that the functional profiles did not exhibit statistically significant separation among the ad libitum feeding group, daytime feeding group, and nighttime feeding group (Figure 2A). However, a statistically significant difference in the Shannon index was observed between the daytime and nighttime feeding groups, whereas no significant difference in the Simpson index was detected across the three feeding groups (Figure 2B). functional profiles using VFDB and secretion system annotations. Compared with ad libitum feeding, daytime feeding showed higher levels of invasion-related functions, enzymatic inactivation modules, and iron-related functions. In contrast, nighttime feeding showed higher levels of immune-evasion–related and adhesion-related functions, especially fimbriae- and pilus-associated traits (Figures 2C–G). These results show that different feeding schedules were linked to different virulence-related functional profiles.

### 3.3 Daytime feeding augments T3SS and T6SS secretion pathway capacity

**Figure 3:**
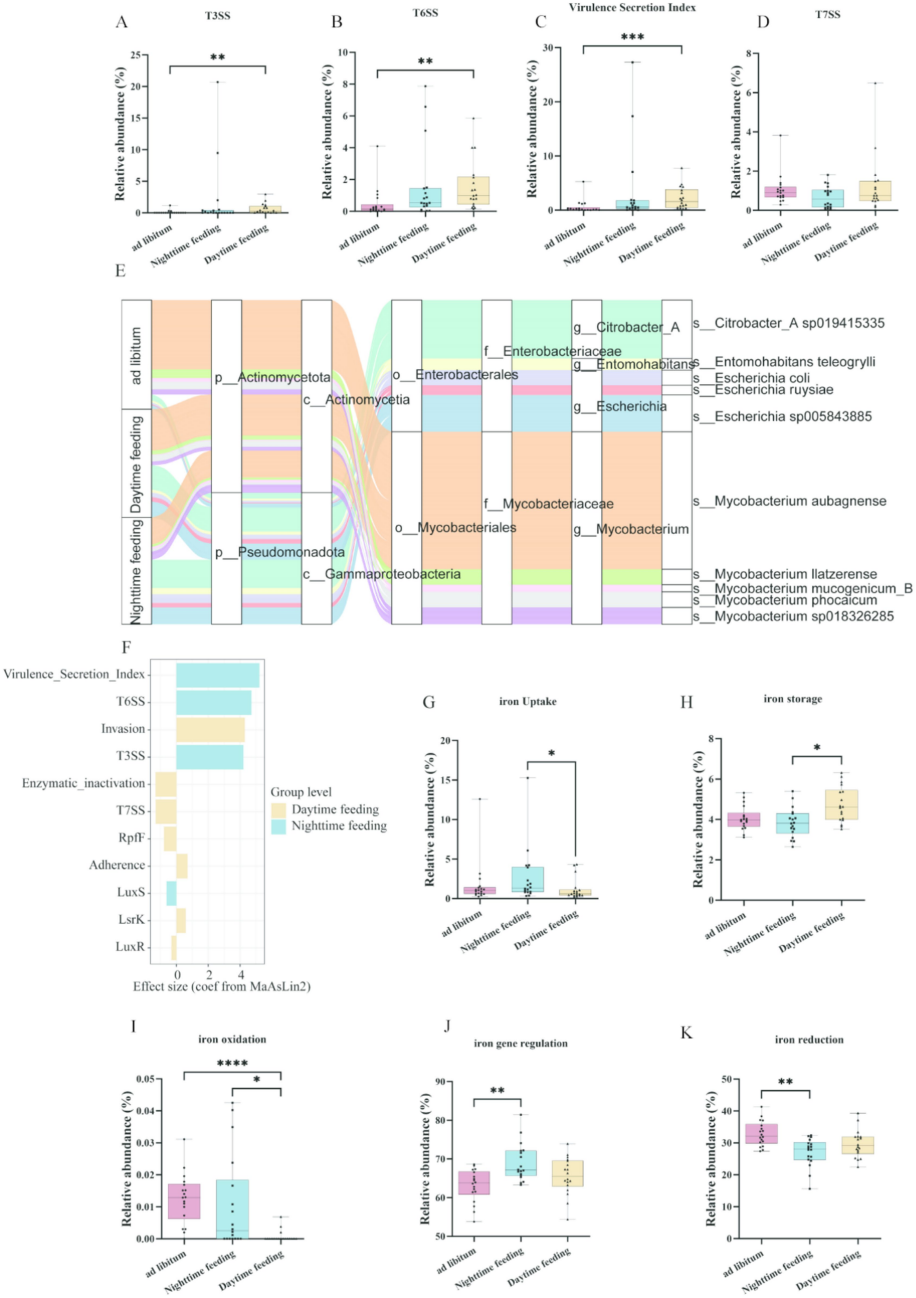
Feeding-dependent changes in microbial secretion systems, contributing taxa, and iron metabolism functions. **(A)** MaAsLin2 analysis of virulence-related functional terms from colonic content metagenomic data. **(B–E)** Relative abundances of T3SS, T6SS, virulence secretion index (VSI), and T7SS inferred from colonic content samples among the *ad libitum*, nighttime, and daytime feeding groups. **(F)** Taxonomic contribution of virulence-associated secretion system functions from colonic content metagenomes. **(G–K)** Differences in iron metabolism functions, including iron uptake, storage, oxidation, regulation, and reduction, inferred from colonic content metagenomic data. *p < 0.05, **p < 0.01, ***p < 0.001; ns, not significant.

To assess changes in secretion systems, we quantified the relative abundance of secretion-related modules. Compared with ad libitum feeding, daytime feeding showed significantly higher levels of T3SS and T6SS secretion system functions (*p* < 0.01; Figures 3A,B). In contrast, T7SS secretion system levels did not change (Figure 3D). This result shows that secretion system changes were specific and not global. The combined virulence secretion index, which included T3SS, T6SS, and effector modules, was highest in the daytime feeding group (*p* < 0.001; Figure 3C). Taxonomic analysis showed that increases in secretion-related functions mainly came from Enterobacteriaceae, including *Escherichia* species, and from *Mycobacterium*-related taxa (Figure 3E). Analysis using MaAsLin2 showed that the feeding pattern was an independent factor linked to secretion module levels, and daytime feeding had the strongest effect (Figure 3F). Iron-related functions, including iron uptake, storage, oxidation, regulation, and reduction, also differed among feeding groups, and several of these functions were higher in the daytime feeding group (Figures 3G–K).

### 3.4 Pathogenic outcome profiles changed without changes in phenotype diversity

**Figure 4:**
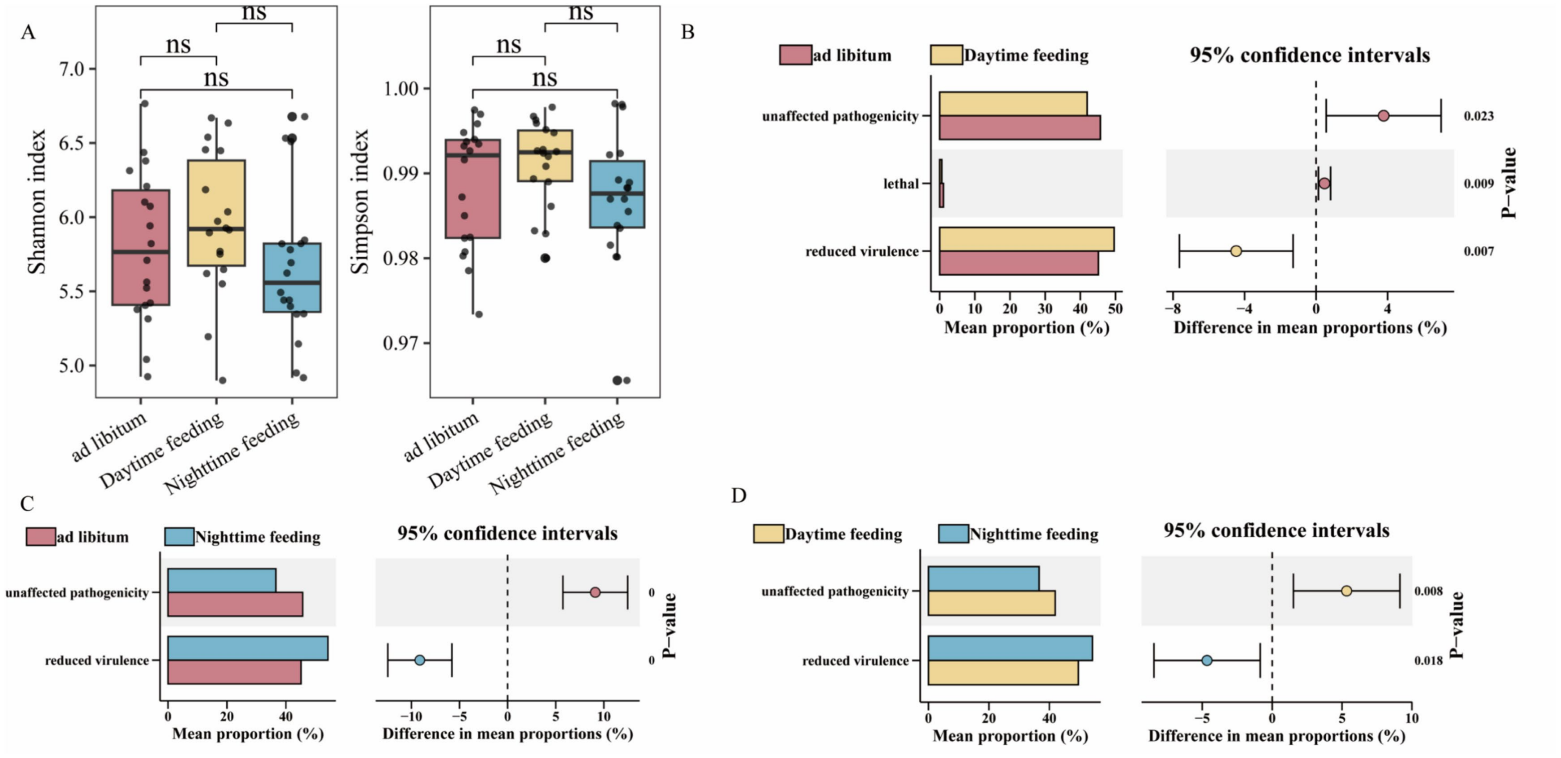
PHI gene diversity and inferred phenotype composition across feeding regimens. **(A)** Comparison of PHI gene α-diversity (Shannon and Simpson indices) calculated from colonic content metagenomic data among the ad libitum, daytime, and nighttime feeding groups. **(B–D)** Differences in inferred PHI phenotype categories, including unaffected pathogenicity, reduced virulence, and lethal traits, based on colonic content samples, across pairwise feeding comparisons: **(B)** Ad libitum vs. daytime feeding; **(C)** Ad libitum vs. nighttime feeding; **(D)** Daytime vs. nighttime feeding. *p < 0.05, **p < 0.01, ***p < 0.001; ns, not significant.

**Figure 5:**
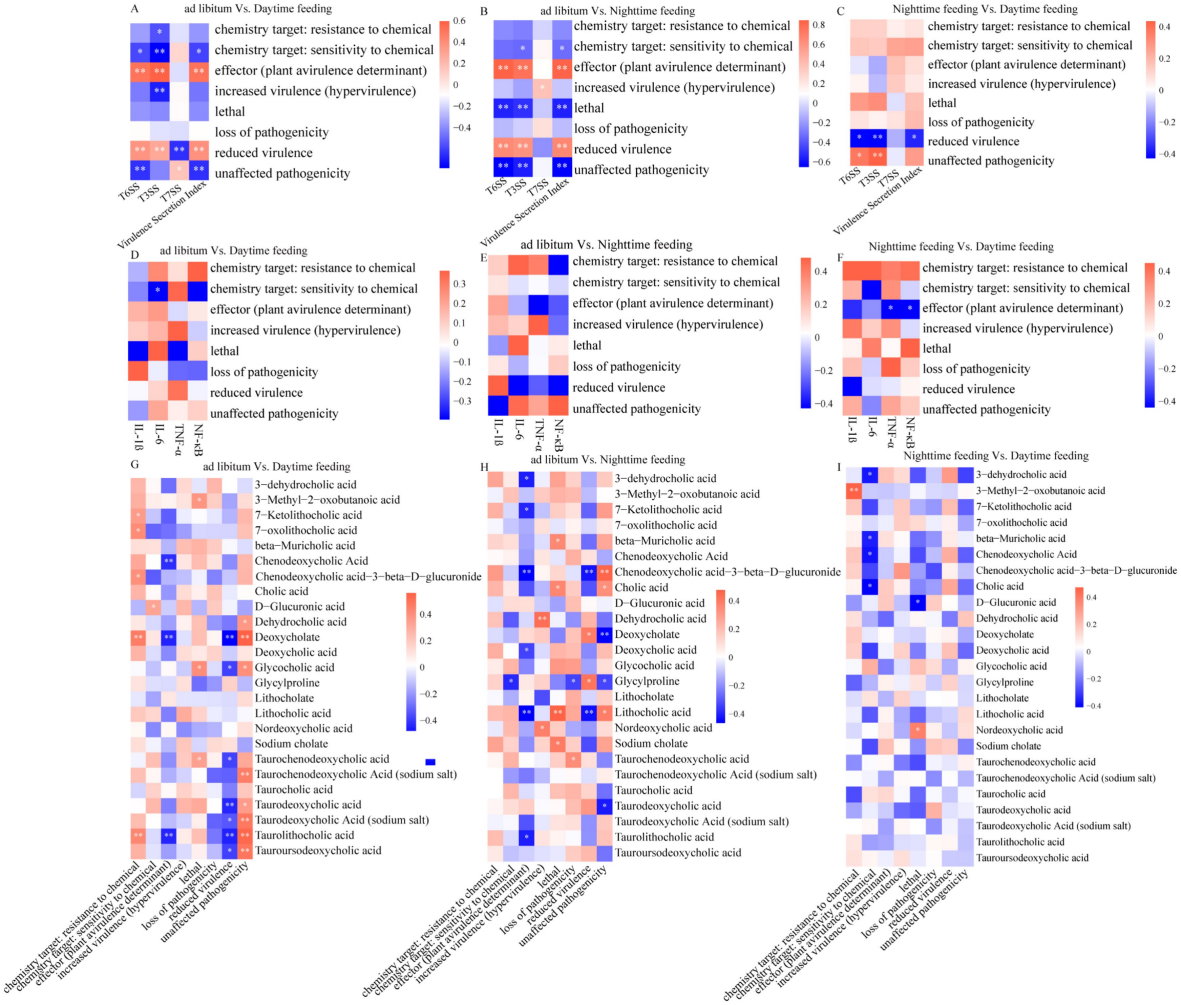
Correlation network linking PHI phenotypes with bacterial virulence secretion systems, host inflammatory responses, and plasma bile acid metabolism under different feeding regimens. **(A–C)** Heatmaps showing Spearman correlations between microbial PHI mutant phenotypic categories and bacterial virulence secretion systems, as well as the VSI. **(D–F)** Heatmaps showing correlations between PHI phenotypes and host inflammatory cytokines. **(G–I)** Correlation heatmaps between PHI phenotypic traits and plasma bile acid metabolites. The color scale represents correlation coefficients (red: positive; blue: negative). ^*^p < 0.05, **p < 0.01, ***p < 0.001; ns, not significant.

To study microbial pathogenic potential, we inferred phenotypes using PHI genes. The *α*-diversity of PHI genes did not differ among feeding groups (*p* > 0.05; Figure 4A). This result shows that overall phenotype diversity remained stable. However, the relative proportions of different phenotypes differed. Daytime feeding showed a lower proportion of “loss-of-pathogenicity” phenotypes. Nighttime feeding showed a higher proportion of reduced-virulence phenotypes (*p* < 0.05; Figures 4B,C). Both time-restricted feeding groups showed lower levels of lethality-related phenotypes than the ad libitum group (Figure 4D). Correlation analysis showed that PHI-based phenotypes were linked to inflammatory markers (NF-κB and IL-1β) and to plasma bile acid levels. These relationships differed across feeding groups (Figure 5).

### 3.5 Coordinated associations among virulence functions, iron metabolism, bile acid profiles, and inflammatory responses

**Figure 6:**
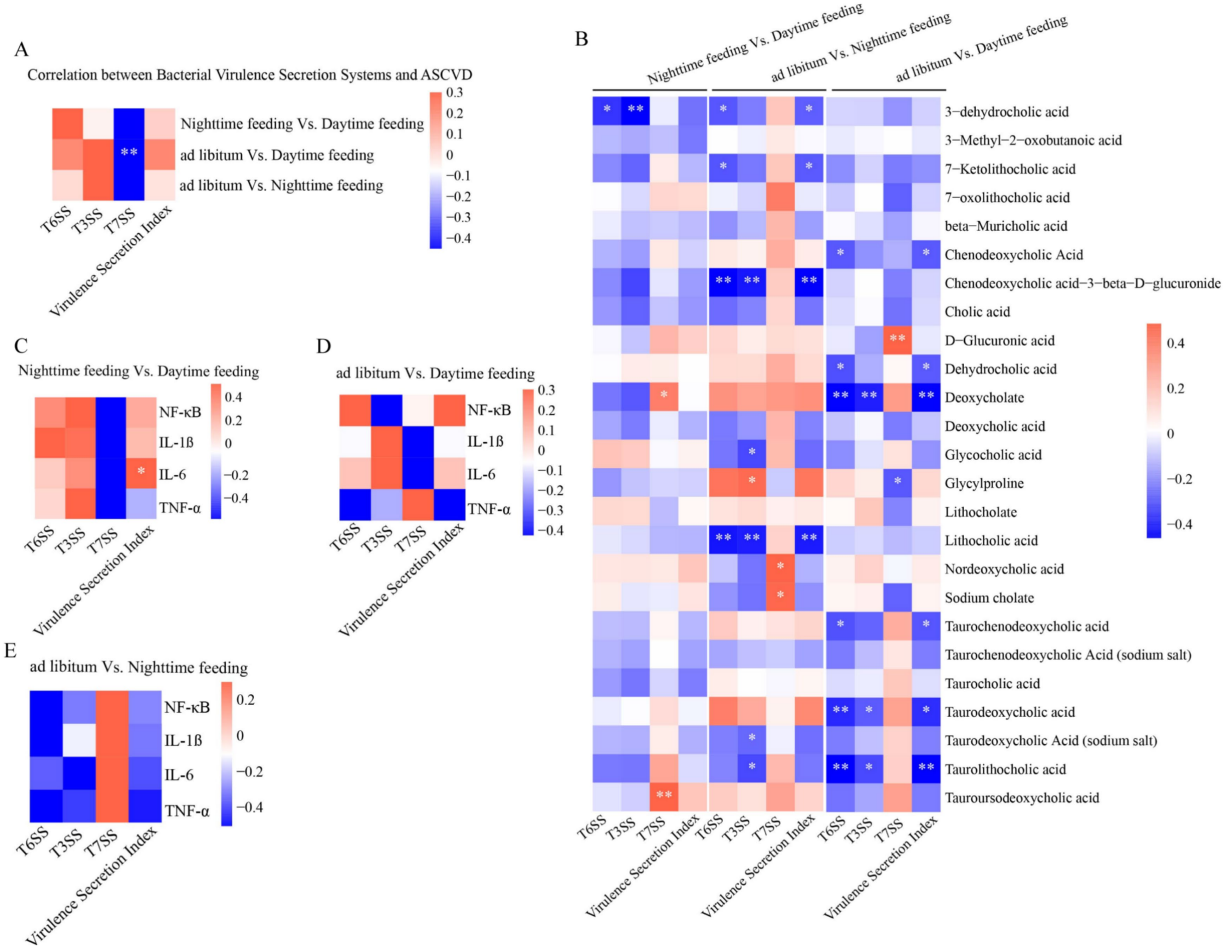
Correlation analyses between bacterial virulence secretion systems and atherosclerotic plaque burden, inflammatory cytokines, and circulating plasma bile acid profiles. **(A)** Heatmap showing spearman correlations between virulence-associated secretion systems and the aortic atherosclerotic lesion area under different feeding conditions. **(B)** Spearman correlation matrix illustrating associations between virulence secretion systems and plasma bile acid species across three pairwise feeding-group comparisons. **(C–E)** Heatmaps showing correlations between virulence factors and inflammatory cytokines (NF-κB, IL-1β, IL-6, and TNF-α) in aortic root cryosections across groups. *p < 0.05, **p < 0.01, ***p < 0.001; ns, not significant.

**Figure 7:**
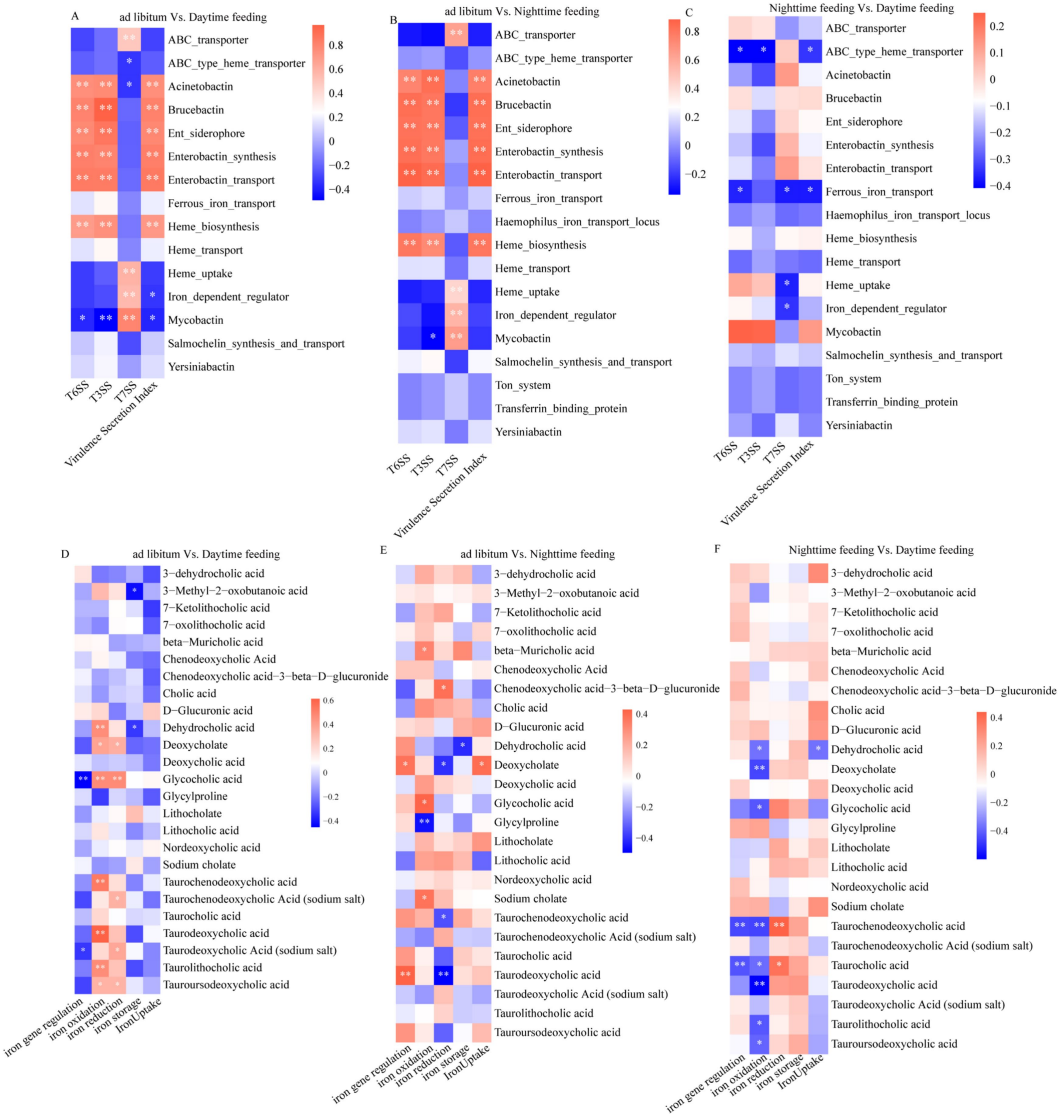
Correlation analysis between bacterial virulence secretion systems and iron acquisition pathways, and between iron metabolism and circulating plasma bile acid profiles under different feeding regimens. **(A–C)** Heatmaps showing Spearman correlations between virulence secretion functions and iron metabolism–related gene categories across three pairwise feeding-group comparisons **(A)** ad libitum vs. daytime feeding; **(B)** ad libitum vs. nighttime feeding; **(C)** nighttime feeding vs. daytime feeding. **(D–F)** Heatmaps showing Spearman correlations between iron acquisition/metabolism pathways and plasma bile acid species across the same three groups. *p < 0.05, **p < 0.01, ***p < 0.001; ns, not significant.

We analyzed correlations among microbial functions, inflammatory markers, and bile acid profiles. These analyses revealed distinct feeding regimen-specific association patterns (Figure 6). T3SS- and T6SS-related scores were positively linked to lesion-linked histological measurements and inflammatory cytokines. At the same time, they were negatively linked to several taurine-conjugated bile acids, including taurochenodeoxycholic acid, which was higher under nighttime feeding. Similarly, iron-acquisition pathway scoring was positively correlated with secretion-related indices but inversely associated with bile acid metabolites previously linked to gut mucosal and metabolic homeostasis (Figure 7).

### 3.6 A connected virulence–iron–QS–ARG network appeared under daytime feeding

**Figure 8:**
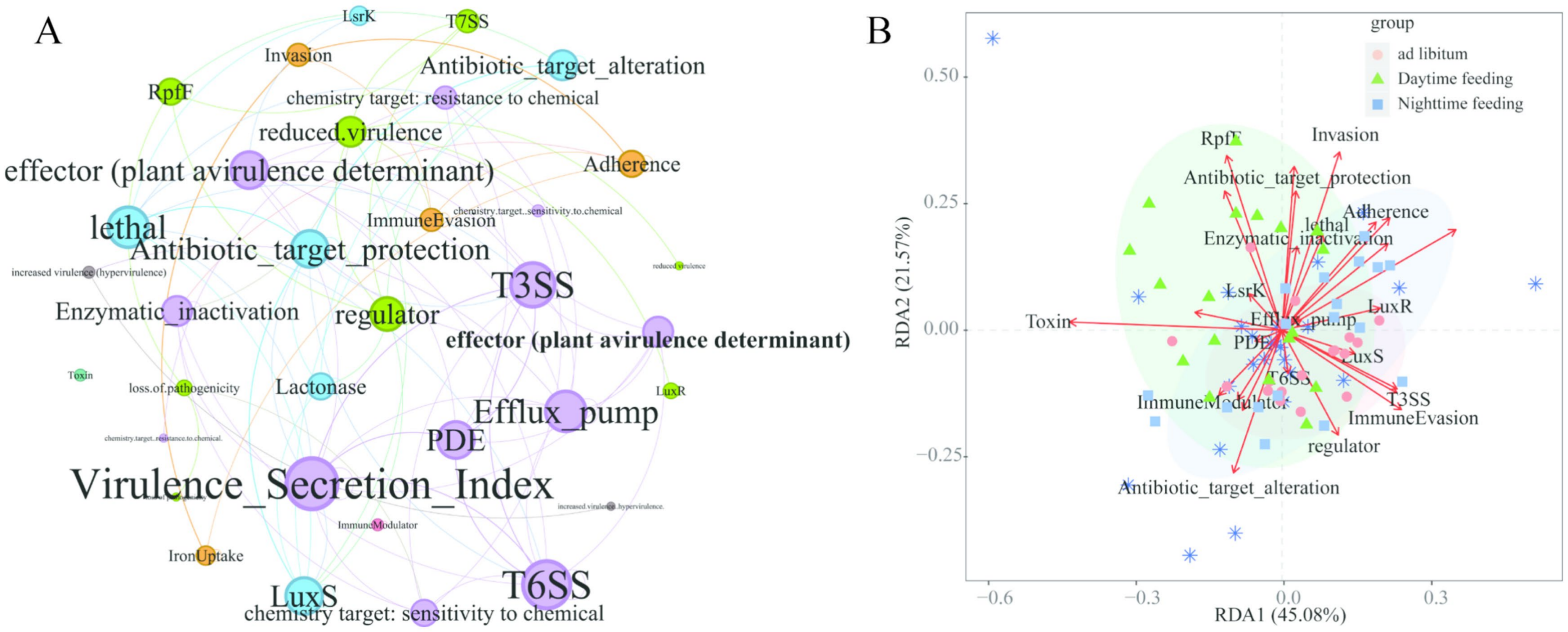
Functional co-occurrence ecological network and RDA ordination linking microbial virulence-associated traits with feeding patterns. **(A)** Functional co-occurrence ecological network. **(B)** Redundancy analysis showing the relationships between microbial functional risk profiles and feeding regimens (*ad libitum*, daytime, and nighttime feeding).

Network-level analysis based on SparCC correlations was used to examine associations among microbial secretion systems, virulence factors, iron metabolism pathways, QS systems, and ARGs. In the daytime feeding group, T3SS- and T6SS-related modules showed strong connections with iron uptake pathways, lux-family quorum-sensing genes (LuxS and LuxR; Supplementary Figure S1), and resistance mechanisms such as efflux pumps and enzymatic inactivation (Supplementary Figure S2), forming a dense network (Figure 8A). In contrast, nighttime feeding showed weaker connections among these functions. Ordination analysis showed that daytime feeding samples grouped with higher levels of secretion, iron, and quorum-sensing functions. Nighttime feeding samples were grouped separately and showed lower overall connectivity. Samples from the ad libitum group were located between these two patterns (Figure 8B).

## 4 Discussion

Accumulating evidence shows that the timing of food intake plays an important role in cardiometabolic health. This role goes beyond the traditional focus on nutrient type or calorie intake (Leech et al., 2024; Raji et al., 2024). In this study, feeding that matched the host circadian phase was linked to a more stable pattern of microbial functions. This pattern supports coordinated metabolic interactions between the host and the microbiota. In contrast, feeding during the inactive circadian phase was linked to higher levels of several virulence-related functions. These included T3SS- and T6SS-related secretion systems, iron uptake pathways, quorum-sensing regulators, and antimicrobial resistance–related functions. These results show that feeding time affects not only microbial structure but also how microbial functions are organized.

Circadian misalignment is linked to metabolic problems, long-term inflammation, and higher cardiovascular risk. This is especially true in people with disrupted daily rhythms, such as shift workers (Zhang et al., 2025a; Manoogian and Panda, 2017; Zhang et al., 2025b; Selingardi et al., 2024). Host circadian clocks normally guide microbial rhythms and gene activity. When this timing is disrupted, changes occur in nutrient availability, oxygen levels, and bile acid flow in the gut. These changes alter the conditions that shape microbial behavior (Heddes et al., 2022; Dantas Machado et al., 2022; Allaband et al., 2021; Bello et al., 2024). Previous studies also show that circadian disruption increases microbial virulence genes and secretion systems (Liu et al., 2025; Borrmann and Rijo-Ferreira, 2024). In line with these findings, our data show that feeding during the inactive phase is linked to higher levels of T3SS and T6SS functions. These changes occur together with increased iron uptake pathways and other pro-inflammatory microbial features. In contrast, feeding during the active phase was linked to microbial functions related to immune adaptation and metabolic cooperation. These patterns suggest that aligned feeding supports cooperation between host and microbes, while misaligned feeding may increase conflict at the host–microbe interface.

A key finding of this study is the identification of a connected group of microbial functions. This group includes secretion systems, iron uptake pathways, quorum-sensing regulators, and antimicrobial resistance features. This group was mainly seen under misaligned feeding conditions. Iron-dependent virulence is well known in pathogenic bacteria. In this process, siderophore-based iron uptake supports toxin secretion and immune escape (Oliveira et al., 2021; Golonka et al., 2019). The presence of iron uptake together with secretion system signals in our network analysis suggests that these functions may be regulated together. This regulation may involve shared factors such as the ferric uptake regulator, Lux-family quorum-sensing proteins, and the RNA-binding protein Hfq.

This functional pattern was also linked to bile acid profiles and inflammatory markers. Bile acid composition in the gut can influence microbial behavior through host signaling pathways and metabolic pressure (Zhang et al., 2025a; Qi et al., 2025). Our analysis showed that higher secretion and iron-related scores were linked to lower levels of several taurine-conjugated bile acids. These bile acids include taurochenodeoxycholic acid, which is known to support gut barrier function and reduce inflammation. These results do not prove cause and effect. Still, they match earlier studies showing that some bile acids can reduce microbial virulence and lower inflammation (He et al., 2022; Sinha et al., 2020; Hu et al., 2021).

Microbial functional diversity remained mostly unchanged across feeding groups. However, clear differences were seen in functional composition and predicted outcomes. This result suggests a shift in functional direction and not a loss of overall function. From an evolutionary view, virulence-related functions are flexible and can be turned on or off. They are not limited to specific microbial groups. This means that microbial risk may arise from changes in functional allocation rather than changes in microbial species. This point may be important for future biomarker development.

Our previous work showed that misaligned feeding lowers TLCA levels and weakens TGR5–ABCA1 signaling. This change promotes vascular inflammation (Zhang et al., 2025a). The current study adds to this finding by showing that misaligned feeding is also linked to higher levels of microbial virulence, iron, and quorum-sensing–related functions. These results suggest that changes in bile acid signaling and microbial function may happen together during circadian disruption. Feeding time appears to influence microbial functions, bile acid balance, and host immune and metabolic signaling at the same time. These combined effects may increase atherosclerotic risk.

This study has several limitations. First, the links between microbial functions and host inflammatory or metabolic markers are based on correlation. They do not prove causality. Second, this study did not include dynamic or rhythmic analysis of microbial functional profiles over a 24-h cycle. Third, although shotgun metagenomics gives broad functional information, we did not perform direct validation experiments. These include gnotobiotic models or targeted manipulation of specific microbial functions. Lastly, metagenomic analysis has technical limits. These include limited strain resolution and incomplete reference databases. These limits may affect the detection of some functions. Future studies should address these issues to better explain how feeding time shapes microbe–host interactions.

## 5 Conclusion

Overall, our results show that feeding time is an important signal that shapes interactions between the gut microbiota and the host. This effect goes beyond changes in microbial species. Feeding that is not aligned with the circadian cycle was linked to higher levels of virulence- and iron-related microbial functions, increased inflammatory signals, and altered bile acid profiles. These findings suggest that matching food intake with the body’s internal clock may help reduce microbe-related inflammation in cardiometabolic disease.

## Data Availability

The data presented in this study are publicly available. This data can be found here: https://www.ncbi.nlm.nih.gov, accession PRJNA1280513.
