# Supplementary material for: Feeding patterns reprogram a gut microbial virulence–iron–quorum sensing functional axis linked to atherosclerotic risk
Source: Front Microbiol. 2026 Jan 21;16:1751844. doi: 10.3389/fmicb.2025.1751844 (PMC12868165; doi:10.3389/fmicb.2025.1751844)
Supplement: Supplementary file 1 [file Data_Sheet_1.pdf]

NIH National Library of Medicine
National Center for Biotechnology Information

Submission Portal

Manage Data > BioProject: PRJNA1280513

BioProject accession	**PRJNA1280513 mouse gut metagenome Raw sequence reads**<br>54 BioSamples<br>54 SRAs
Status	✔ **Released**
Release date	2025-12-19
Created	2025-06-22 07:54
Updated	2025-12-19 18:17
Title	mouse gut metagenome Raw sequence reads<br>Edit
Description	Exploring the Differential Changes in Intestinal Microbial Composition and Function of ApoE-/- Mice under Different Feeding Patterns<br>Edit
Sample scope	Monoisolate
Locus tag prefixes	LTP: ACR814 / BioSample accession:
Organism	mouse gut metagenome<br>Taxonomy ID: 410661
Grants	Add
Publications	Add

SRA (54) BioSample (54)

Edit metadata | Request data removal

Select data using the checkboxes below to edit metadata or request data removal

Request Completed: Change BioProject PRJNA1280513 release date to 2025-12-19 安全浏览模式

发件人：sra<sra@ncbi.nlm.nih.gov> (定制我的回信邮箱域名，对外沟通更显专业，立即定制)

收件人：我<zhanghejt@163.com>

时　间：2025年12月19日 18:18 (星期五)

只显示原文 | 只显示译文 | 对照显示 | 您还可以选择 AI翻译，查看更精准更流畅的译文

Dear He zhang,

This email confirms that NCBI has completed your request to release this BioProject on 2025-12-19.

BioProject: PRJNA1280513

Please see our documentation if you need to request a status change https://www.ncbi.nlm.nih.gov/sra/docs/request-status-change/ or make modifications https://www.ncbi.nlm.nih.gov/sra/docs/submitupdate/. Please contact sra@ncbi.nlm.nih.gov if it has been 2 weeks and you do not see your data public at https://www.ncbi.nlm.nih.gov/bioproject/PRJNA1280513.

If you requested release for Accessions not listed above, you will receive a separate message about that release.

If there are other data that you want to release now, for example assembled genomes, then please send your request to us at genomes@ncbi.nlm.nih.gov with the relevant accession numbers.

Note that BioProjects and BioSamples are triggered for release when their linked sequence data, for example SRA or assembled genomes, are public. However, the release of a BioProject or BioSample will not trigger the release of linked data.

Thank you!

Manage Data

Type in data information to filter accessions | Search

Linked to PRJNA1280513

All (108) BioProject (0) BioSample (54) **SRA (54)**

Filter by status:

Released (54) | To be released | Processing | Error | Suppressed | Withdrawn | Discontinued

Clear all

Filter by date:

From date YYYY-MM-DD To date YYYY-MM-DD

Accession	Title	BioProject	BioSample	Library ID	Files	Status	Release date	Updated
SRR34092318	Metagenome of Colonic Contents in ApoE-/- Mice	PRJNA1280513	SAMN49518810	N-ZT20-2	N-ZT20-JS2-1_R1.fq.gz<br>N-ZT20-JS2-1_R2.fq.gz	✔ Released	2025-12-19	18:17
SRR34092317	Metagenome of Colonic Contents in ApoE-/- Mice	PRJNA1280513	SAMN49518811	N-ZT20-3	N-ZT20-JS3-1_R1.fq.gz<br>N-ZT20-JS3-1_R2.fq.gz	✔ Released	2025-12-19	18:17
SRR34092315	Metagenome of Colonic Contents in ApoE-/- Mice	PRJNA1280513	SAMN49518765	A-ZT08-2	A-ZT8-JS2-1_R1.fq.gz<br>A-ZT8-JS2-1_R2.fq.gz	✔ Released	2025-12-19	18:17
SRR34092316	Metagenome of Colonic Contents in ApoE-/- Mice	PRJNA1280513	SAMN49518764	A-ZT08-1	A-ZT8-JS1-1_R1.fq.gz<br>A-ZT8-JS1-1_R2.fq.gz	✔ Released	2025-12-19	18:17
SRR34092313	Metagenome of Colonic Contents in ApoE-/- Mice	PRJNA1280513	SAMN49518767	A-ZT12-1	A-ZT12-JS1-1_R1.fq.gz<br>A-ZT12-JS1-1_R2.fq.gz	✔ Released	2025-12-19	18:17
SRR34092314	Metagenome of Colonic Contents in ApoE-/- Mice	PRJNA1280513	SAMN49518766	A-ZT08-3	A-ZT8-JS3-1_R1.fq.gz<br>A-ZT8-JS3-1_R2.fq.gz	✔ Released	2025-12-19	18:17
SRR34092350	Metagenome of Colonic Contents in ApoE-/- Mice	PRJNA1280513	SAMN49518781	D-ZT04-3	D-ZT4-JS3-1_R1.fq.gz<br>D-ZT4-JS3-1_R2.fq.gz	✔ Released	2025-12-19	18:17
SRR34092349	Metagenome of Colonic Contents in ApoE-/- Mice	PRJNA1280513	SAMN49518782	D-ZT08-1	D-ZT8-JS1-1_R1.fq.gz<br>D-ZT8-JS1-1_R2.fq.gz	✔ Released	2025-12-19	18:17
SRR34092348	Metagenome of Colonic Contents in ApoE-/- Mice	PRJNA1280513	SAMN49518783	D-ZT08-2	D-ZT8-JS2-1_R1.fq.gz<br>D-ZT8-JS2-1_R2.fq.gz	✔ Released	2025-12-19	18:17
SRR34092347	Metagenome of Colonic Contents in ApoE-/- Mice	PRJNA1280513	SAMN49518784	D-ZT08-3	D-ZT8-JS3-1_R1.fq.gz<br>D-ZT8-JS3-1_R2.fq.gz	✔ Released	2025-12-19	18:17
SRR34092345	Metagenome of Colonic Contents in ApoE-/- Mice	PRJNA1280513	SAMN49518786	D-ZT12-2	D-ZT12-JS2-1_R1.fq.gz<br>D-ZT12-JS2-1_R2.fq.gz	✔ Released	2025-12-19	18:17
SRR34092346	Metagenome of Colonic Contents in ApoE-/- Mice	PRJNA1280513	SAMN49518785	D-ZT12-1	D-ZT12-JS1-1_R1.fq.gz<br>D-ZT12-JS1-1_R2.fq.gz	✔ Released	2025-12-19	18:17
SRR34092343	Metagenome of Colonic Contents in ApoE-/- Mice	PRJNA1280513	SAMN49518761	A-ZT04-1	A-ZT4-JS1-1_R1.fq.gz<br>A-ZT4-JS1-1_R2.fq.gz	✔ Released	2025-12-19	18:17
SRR34092344	Metagenome of Colonic Contents in ApoE-/- Mice	PRJNA1280513	SAMN49518787	D-ZT12-3	D-ZT12-JS3-1_R1.fq.gz<br>D-ZT12-JS3-1_R2.fq.gz	✔ Released	2025-12-19	18:17
SRR34092342	Metagenome of Colonic Contents in ApoE-/- Mice	PRJNA1280513	SAMN49518788	D-ZT16-1	D-ZT16-JS1-1_R1.fq.gz	✔ Released	2025-12-19	18:17
